# Intrinsic brain connectivity alterations despite intact pain inhibition in subjects with neuropathic pain after spinal cord injury: a pilot study

**DOI:** 10.1038/s41598-023-37783-w

**Published:** 2023-07-24

**Authors:** Vincent Huynh, Robin Lütolf, Jan Rosner, Roger Luechinger, Armin Curt, Spyridon Kollias, Lars Michels, Michèle Hubli

**Affiliations:** 1 Spinal Cord Injury Center, Balgrist University Hospital, University of Zurich, Forchstrasse 340, 8008 Zurich, Switzerland; 2 Department of Neuroradiology, Clinical Neuroscience Center, University Hospital Zurich & University of Zurich, Zurich, Switzerland; 3 Department of Neurology, University Hospital Bern, Inselspital, University of Bern, Bern, Switzerland; 4 Institute for Biomedical Engineering, University and ETH Zurich, Zurich, Switzerland; 5 Present Address: Spinal Cord Injury Center, Balgrist University Hospital, University of Zurich, Forchstrasse 340, 8008 Zurich, Switzerland

**Keywords:** Neuroscience, Neuropathic pain

## Abstract

Endogenous pain modulation in humans is frequently investigated with conditioned pain modulation (CPM). Deficient pain inhibition is a proposed mechanism that contributes to neuropathic pain (NP) after spinal cord injury (SCI). Recent studies have combined CPM testing and neuroimaging to reveal neural correlates of CPM efficiency in chronic pain. This study investigated differences in CPM efficiency in relation to resting-state functional connectivity (rsFC) between 12 SCI-NP subjects and 13 age- and sex-matched healthy controls (HC). Twelve and 11 SCI-NP subjects were included in psychophysical and rsFC analyses, respectively. All HC were included in the final analyses. Psychophysical readouts were analysed to determine CPM efficiency within and between cohorts. Group differences of rsFC, in relation to CPM efficiency, were explored with seed-to-voxel rsFC analyses with pain modulatory regions, e.g. ventrolateral periaqueductal gray (vlPAG) and amygdala. Overall, pain inhibition was not deficient in SCI-NP subjects and was greater in those with more intense NP. Greater pain inhibition was associated with weaker rsFC between the vlPAG and amygdala with the visual and frontal cortex, respectively, in SCI-NP subjects but with stronger rsFC in HC. Taken together, SCI-NP subjects present with intact pain inhibition, but can be differentiated from HC by an inverse relationship between CPM efficiency and intrinsic connectivity of supraspinal regions. Future studies with larger cohorts are necessary to consolidate the findings in this study.

## Introduction

Dysfunction of endogenous pain modulation is often reported in chronic pain conditions^1–5^. In humans, pain modulatory function is commonly assessed with conditioned pain modulation (CPM), a psychophysical paradigm that involves the application of a noxious *test stimulus* (TS) which is modulated by another remotely applied noxious *conditioning stimulus* (CS)^6–8^. Recent studies have observed that dysfunction of endogenous pain modulation, i.e. impaired pain inhibition/diminished CPM efficiency, could be contributing to the development and maintenance of neuropathic pain (NP) after spinal cord injury (SCI)^9–11^. Two studies have reported deficient pain inhibition in subjects with NP after SCI (SCI-NP) compared to those without NP and healthy controls (HC)^9,10^. In contrast, Gruener et al.^12^ showed intact CPM efficiency, i.e. pain inhibition, in SCI-NP, SCI without NP as well as HC. Furthermore, a longitudinal study observed a preservation of pain inhibition in SCI-NP over time, even though CPM efficiency declined from time of hospital admission to discharge^11^. With regard to NP prediction, a lack of pain inhibition tested at the level of the injury at 1.5 months post injury was predictive of greater pain severity at 24 months following SCI^12^. CPM efficiency has also been related to NP characteristics (e.g. intensity and extent), with studies observing higher number of painful body regions^10^ and more intense NP^9,13^ correlated with deficient pain inhibition in SCI-NP subjects. A more recent longitudinal study however observed more intense NP correlated with greater pain inhibition^11^. Hence, altered pain modulation can be related to the occurrence of NP and its severity in subjects with SCI, though current observations remain contradictory and require further exploration.

CPM is known to be partly mediated by descending brainstem pathways via a spino-bulbo-spinal loop^14–19^. Growing evidence suggests that intrinsic activity, i.e. resting-state functional connectivity (rsFC), between cerebral regions play a role in individual CPM efficiency and are abnormal in chronic pain conditions^20–22^. For instance, deficient pain inhibition has been related to stronger rsFC between brainstem regions of the descending pain modulatory system (e.g. ventrolateral periaqueductal gray (vlPAG) and rostro-ventral medulla) in fibromyalgia^22^, and areas involved with cognitive-affective components of pain (e.g. frontal cortex and amygdala) in chronic neck pain sufferers^20^. Although intact pain inhibition was observed in migraineurs psychophysically, the association between individual CPM efficiency and rsFC amongst hubs of the default-mode network in migraineurs was found to be altered compared to HC^21^. Specifically, stronger rsFC between the anterior cingulate cortex (ACC), prefrontal cortex and the precuneus were related to more efficient CPM (i.e. stronger pain inhibition) in HC but this relationship was weakened in migraineurs. More efficient CPM in migraineurs was also found to be related to stronger rsFC between the anterior insula and angular gyrus^21^. These observations indicate that pain could alter the interplay between individual pain modulation and underlying neural activity in regions involved with top-down (e.g. vlPAG) and bottom-up (e.g. insula) pain processing, yet this relationship remains unknown in SCI-NP.

Thus, the aim of this study was to explore the pain modulatory capacity in SCI-NP and detail its relationship with the neural resting-state connectivity of the brain and NP characteristics. To this end, we recruited SCI-NP subjects and HC who underwent CPM testing, resting-state functional magnetic resonance imaging (fMRI) and pain characterisation. As studies report inconsistent psychophysical findings with regard to CPM efficiency in SCI-NP^9–12^, we decided to use an exploratory approach to investigate the potentially altered pain inhibition in relation to rsFC in SCI-NP subjects compared to HC.

## Methods

### Subjects

Thirteen SCI-NP subjects and 13 age- and sex-matched HC were recruited for this study (Table 1). SCI subjects were recruited from the Spinal Cord Injury Center at Balgrist University Hospital and the Swiss Spinal Cord Injury Cohort Study database, while HC were recruited via online flyer advertisements. The inclusion criteria for SCI subjects were: (1) the presence of NP by the current diagnostic criteria^23^, (2) 18–80 years old, (3) non-cervical SCI, i.e. thoracic or high lumbar level of injury, (4) no contraindications for MRI, and v) no history or presence of other neurological, psychological or medical conditions (e.g. traumatic brain injury, diabetes, cancer). The inclusion criteria for HC were: (1) 18–80 years old, (2) no neurological or psychiatric conditions, (3) no history of chronic pain or pain during participation and (4) no intake of psychoactive medication. The 13 age- and sex-matched HC were selected from a primary cohort of 40 HC^24^. The psychophysical and neuroimaging data obtained from this cohort are reported separately^24^. The matching process of the 13 HC was performed by a secondary investigator (MH) and the primary assessor (VH) was blinded from this selection procedure. Written informed consent was acquired from all subjects prior to the assessments. All procedures described are in accordance with the Declaration of Helsinki and the study has been approved by the local ethics board ‘Kantonale Ethikkommission Zürich, KEK’ (EK-04/2006, PB_2016-02051, clinicaltrial.gov number: NCT02138344).

**Table 1:** Summary of subjects’ characteristics.

Characteristics	SCI-NP (n = 12)	HC (n = 13)
Demographics		
Age (years)	59 (38–66)	53 (37–70)
Sex (F/M)	1/11	1/12
Handedness (R/L)	11/1	12/1
Neurological level of lesion	Th1–L1	–
AIS (A–D)	6 A, 1 B, 1 C, 4 D	–
Time since injury (years)	17 (7–39)	–
NP characteristics		
NP intensity (NRS)	4.3 (1.4–6.5)	–
NP extent (%)	6.8 (1.0–35.5)	–
Questionnaires		
PCS (0–52)	11 (1–20)	11 (0–23)
BDI (0–63)	3 (0–14)	2 (0–17)
Thermal thresholds (°C)		
WDT	35.8 ± 1.1	36.1 ± 1.2
HPT	46.1 ± 3.6	43.6 ± 2.8
CPM effect (NRS)		
TS-CPM–TS-Sham	− 0.92 ± 1.08	− 0.41 ± 1.11
CPM profiles (n)		
Inhibitor	10	8
Non-responder	–	–
Facilitator	2	5

Information is presented as mean (± standard deviation) or median (range). CPM effect: difference between averaged pain ratings during TS-CPM and TS-Sham. Positive numbers describe a facilitatory CPM effect (facilitator), whilst negative numbers describe an inhibitory CPM effect (inhibitor).

AIS, ASIA Impairment Scale (A, sensorimotor complete; C–D, motor incomplete); BDI, Beck Depression Inventory; CPM, conditioned pain modulation; CS, conditioning stimulus; F, female; HPT, heat pain threshold; L, left; M, male; NRS, numerical rating scale; PCS, Pain Catastrophising Scale; R, right; TS, test stimulus; TS-CPM, test stimulus under CPM; TS-Sham, test stimulus under Sham; WDT, warm detection threshold.

### NP characterisation in SCI subjects

Diagnosis of NP in SCI subjects adhered to current recommendations which includes the presence of a neurological lesion of the spinal cord^23^. Overall, presence of typical sensory signs and symptoms is required and the area of pain needed to follow a plausible neuroanatomical distribution with respect to the lesion level.

To assess the intensity and spatial extent of NP, each subject completed a pain drawing prior to MRI scanning^25^. The drawing consists of a body schematic (front and back) where subjects were asked to draw the current pain location, distribution alongside its intensity indicated on an 11-point numerical rating scale (NRS) (0, ‘no pain’ to 10, ‘worst pain imaginable’). A standardised scheme presenting the full body dermatomes was laid on the pain drawing in order to delineate at- and below-level NP, which was distinguished as pain within or below three dermatomes of the lesion, respectively. After identifying the NP, each affected area was highlighted and quantified into an overall percentage of NP extent (%), which was determined by the sum of total pixel count from both front and back of subjects’ digitalised pain drawings divided by the total pixel count of the body schematic. NP extent as characterised by these pain drawings has been shown to have excellent inter-session reliability^25^. Averaged NP intensity of each SCI subject was obtained by calculating the sum of NP intensity divided by the amount of particular regions that the subjects rated as painful.

### Study design

All subjects completed questionnaires, thermal thresholds and a familiarisation procedure for the heat stimuli of the following CPM paradigm. The Pain Catastrophising Scale (PCS)^26^ and Beck Depression Inventory version II (BDI)^27^ were filled out in order to provide psychological outcome measures of each subject. To ensure sensory integrity of the tested area, i.e. the volar forearm as an area above the neurological level of injury in our paraplegic subjects, thermal thresholds were examined according to the quantitative sensory testing protocol of the German Research Network on Neuropathic Pain^28^. Warm detection and heat pain thresholds (WDT and HPT, respectively) were assessed with the PATHWAY Pain & Sensory Evaluation system using the 3 cm × 3 cm square ATS thermode (Medoc Ltd, Ramat Yishai, Israel) at the volar forearm (half way between the wrist and the cubital fossa) on the side of the dominant hand. Each trial began with a baseline thermode temperature of 32 °C and increased at a rate of 1 °C per second. Subjects were required to click the response unit as soon as they perceived a change in temperature (WDT) or at the initial sensation of pain (HPT). Thermal thresholds were determined by averaging three trials of individual stimuli. Safety cut-off temperatures for WDT and HPT were set at 55 °C. All subjects were blinded from the operator screen during threshold testing. After obtaining thermal thresholds, subjects were acquainted with the instructions of MRI acquisition and the CPM procedure including the pain rating process.

### Conditioned pain modulation

**Figure 1:**
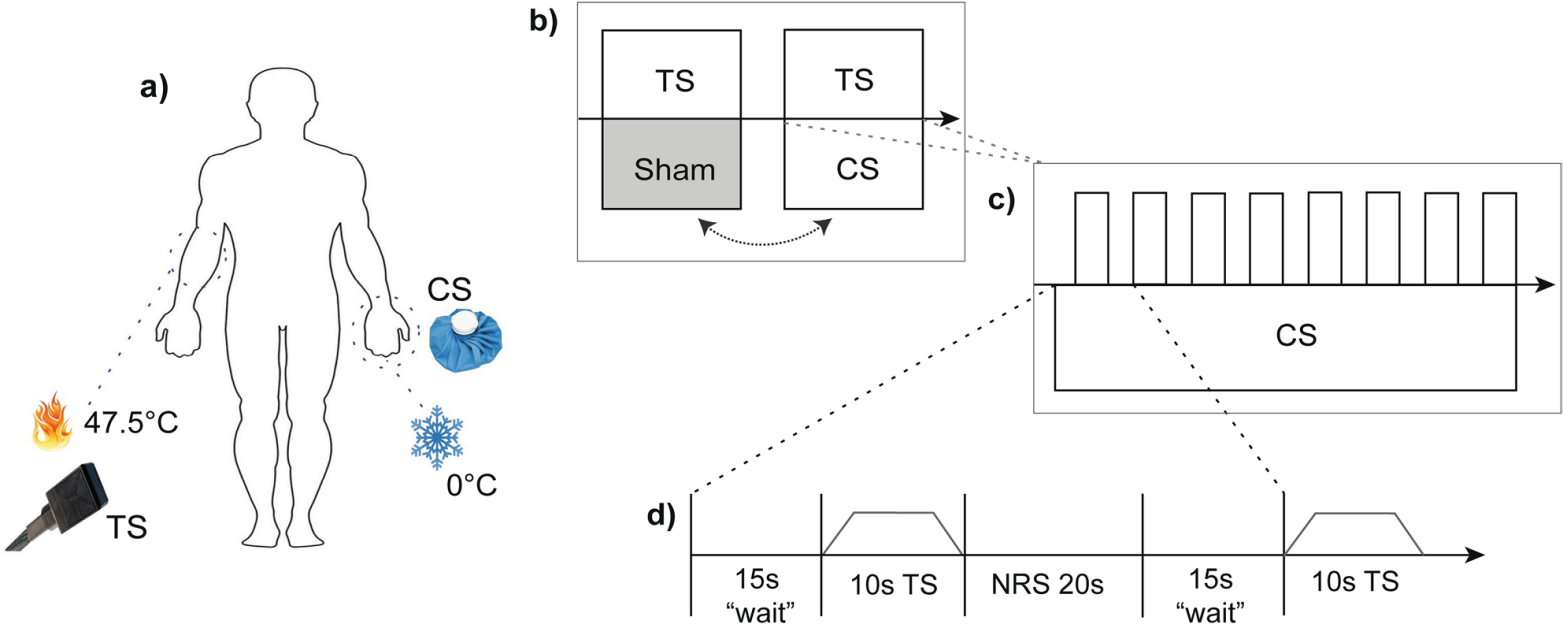
Conditioned pain modulation paradigm. Experimental design of the CPM paradigm performed within the MRI scanner. (**a**) Parallel CPM paradigm was performed with thermal stimuli at the volar forearm and hand. A thermode ramping up to 47.5 °C acted as the TS and two ice bags at 0 °C on the contralateral hand acted as the CS. (**b**) two randomised CPM conditions were used to assess endogenous pain modulation in each subject. One condition included the application of the genuine CS whilst the other involved the application of two water filled bags at skin temperature (~ 32 °C) which acted as the sham CS. (**c**, **d**) Each condition consisted of eight TS applied to the dominant volar forearm which started with a waiting period of 15 s followed by the TS that lasted 10 s. Following each TS, a 20 s period was allocated to obtain the pain perception of TS and CS on an NRS. The inter-stimulus interval between each TS was 35 s. CPM, conditioned pain modulation; CS, conditioning stimulus; MRI, magnetic resonance imaging; TS, test stimulus. With permission from Huynh et al.^24^.

The CPM paradigm is summarised in Fig. 1. Inside the MRI scanner, a parallel CPM paradigm with two conditions was performed with each subject: (1) TS with a CS (TS-CPM) and (2) TS with a sham condition (TS-Sham). The two conditions were randomised across subjects in a balanced fashion and lasted 6:10 min with a five-minute break in between. The TS was applied with a 3 cm × 3 cm square ATS thermode (Medoc Ltd, Ramat Yishai, Israel) attached to the volar forearm (half way between the wrist and the cubital fossa) of the subject's dominant hand. Per condition, eight TS were applied with an inter-stimulus interval of 35 s with each TS having a fixed target temperature of 47.5 °C and lasted a total of 10 s including ramp time (2.5 s ramp up, 5 s plateau, 2.5 s ramp down)^29^. Between each condition, the position of the thermode was slightly shifted to alleviate sensitisation effects. Following each TS, subjects had 20 s to rate their perceived pain of the TS and CS (10 s each) on a NRS (0, ‘no pain’ to 10, ‘worst pain imaginable’). The NRS was projected on the NordicNeuroLab 32″ screen (NordicNeuroLab, Norway and USA, https://www.nordicneurolab.com) and the subjects rated using a manual response unit placed in their dominant hand. This unit was programmed to move a box either up or down the NRS per click of the allocated button, i.e. if subjects perceived the pain of the TS to be a ‘five’ subjects clicked button 1 five times to move the box up the NRS. Subjects were always prompted to rate the TS followed by rating of the CS. Ratings of the CS were taken during both actual and sham conditions. The CS consisted of two ice bags covering the non-dominant hand for the whole duration of the condition. Each ice bag contained ~ 600 g of ice and 250 ml of water guaranteeing a stable temperature of 0 °C. Pain rating of the CS from pilot data (n = 5) indicated it being an appropriate noxious stimulus with an averaged pain perception of NRS 7.0 (range = 6.3–8.0). As recommended by Yarnitsky et al.^8^, an appropriate noxious stimulus is determined at an intensity of 4/10. For the sham condition, two bags with water at skin temperature (~ 32 °C) were used. Water temperature was measured at the beginning with a thermometer. This CPM paradigm was adapted from a previous study utilising similar ice bags, noxious stimuli and block timings^29^. In particular, we applied the CS at the hand rather than the whole leg to prevent autonomic dysreflexia in our SCI-NP cohort^30^.

### MRI data acquisition

All subjects’ MRI images were obtained with the 3.0 Tesla Philips Ingenia system (Philips Medical Systems, Best, the Netherlands) using a 32-channel Philips head coil. 3D T1-weighted structural images were acquired with a Turbo Field Echo sequence with the following parameters: repetition time (TR), 8.1 ms; echo time (TE), 3.7 ms; flip angle (FA), 8°; number of slices, 160; slice thickness, 1 mm; field of view (FOV), 240 × 240 × 160 mm^3^; matrix, 240 × 240 and isotropic voxel 1 × 1 × 1 mm^3^. Scan time was a total of 4:53 min. Resting-state functional images were acquired with an echo-planar-imaging sequence with the following parameters: TR, 2000 ms; TE, 30 ms; FA, 78°; number of slices, 36; FOV, 220 × 136 ×  220 mm^3^; matrix, 72 × 74; voxel size, 3.0 × 3.0 × 3.0 mm; reconstructed voxel size, 1.72 × 1.72 × 3 mm^3^ and a scan time of 5:00 min. During the resting-state acquisition, subjects were instructed to relax and fixate on a motionless cross, projected on a NordicNeuroLab 32″ screen (NordicNeuroLab, Norway and USA, https://www.nordicneurolab.com). To minimise motion, cushions were placed around each subject’s head. Structural and resting-state functional MRI data acquisition was performed prior to the CPM paradigm. This sequence and paradigm was used in a previous study^24^. Task-related functional MRI data was acquired during the CPM paradigm, but is not reported here.

### Data analyses

#### CPM psychophysics

For each subject, the CPM effect was calculated as the averaged pain ratings of the eight TS during the TS-Sham condition, subtracted from the averaged pain ratings of the eight TS during the TS-CPM condition (CPM effect = averaged pain ratings of TS [TS-CPM]−averaged pain ratings of TS [TS-Sham]). This provided an overall sham-corrected CPM effect score with negative numbers denoting pain inhibition and positive numbers denoting pain facilitation^8^. Individual CPM effect scores were used in the neuroimaging analyses to investigate brain correlates of CPM effect.

Statistical analyses were performed with the Statistical Package for the Social Sciences (SPSS, version 24). To test normality of subject characteristics and CPM psychophysical readouts, histograms, Q–Q plots and Shapiro–Wilk tests were applied. For non-normally distributed variables non-parametric tests were used. Within each cohort, overall CPM effect was tested with a dependent two-tailed t-test between the averaged pain ratings of the TS in each condition (TS-CPM vs TS-Sham). One-sided T-tests were performed to test the significance of overall CPM effect against 0 for each group.

Correlation analyses were used to test relationships between subjects’ age, WDT, HPT, BDI and PCS scores and pain ratings of the CS with individual CPM effect. Between-group differences in demographics, pain ratings and CPM effect were tested with independent two-tailed t-tests and chi-square tests (for categorical variables). In SCI-NP subjects only, partial correlations were implemented to assess associations between CPM effect and NP characteristics, i.e. NP intensity and extent with age, sex, with PCS and BDI scores as covariates of no interest. Further, we investigated group differences of CPM effect between the SCI-NP subjects on medication vs those not on medication with an independent t-test. Results were deemed significant at p < 0.05.

#### Pre-processing for neuroimaging analysis

Structural T1-weighted images and resting-state fMRI images were pre-processed using Statistical Parametric Mapping (SPM12) software (Wellcome Department of Imaging Neuroscience, London, United Kingdom: (http://www.fil.ion.ucl.ac.uk/spm/) implemented in MATLAB 2017a (The Mathworks, Inc, Natick, MA). Prior to pre-processing, structural and functional images of each subject were realigned and centered to the anterior commissure (Montreal Neurological Institute (MNI) coordinates; MNI = 0, 0, 0) using the SPM12 display function.

Structural scans were segmented into grey matter, white matter and cerebrospinal fluid maps using the New Segment tool^31^. Functional images were pre-processed as follows: realignment (head motion correction), centering (to anterior commissure, MNI co-ordinates = 0,0,0), slice-timing correction (ascending), outlier detection and scrubbing (using ARtifact detection Tools) during the denoising step^32,33^, MNI normalisation and smoothing with a 6 mm Gaussian full width at half maximum (FWHM). The pre-processing steps generated interpolated 2 × 2 × 2 mm^3^ resolution images for the analyses. Head motion during the resting-state scan was assessed with the three translational and rotational dimensions for each scan. Subjects whose mean head motion during the functional scan exceeded + 1.5 mm for translation and/or 1° for rotation were removed from rsFC analyses. During the denoising step, normalisation of voxel-to-voxel connectivity values were performed in addition to linear detrending and subjects that showed normally distributed data after denoising were included for rsFC analyses.

#### Seed-to-voxel rsFC analyses

Resting-state functional MRI data was analysed with the CONN toolbox (CONN 18b; www.nitrc.org/projects/conn)^34^. CONN utilises a component-based noise correction method (CompCor) which increases selectivity, sensitivity and permits a higher degree of inter-scan reliability^35^. A band-pass filter of 0.01–0.1 Hz was applied to remove linear drift artefacts and high-frequency noise. CONN also accounts for outlier data points and movement time courses as nuisance regressors. For each subject, the six motion parameters, activity from segmented white matter and cerebrospinal fluid maps were included as regressors of no interest, thereby reducing noise and signal unlikely to reflect neuronal activity related to functional connectivity.

To test between-group differences in seed-to-voxel rsFC and the relationship between CPM effect and seed-to-voxel rsFC a general linear model was implemented. To this end, a one-way ANCOVA covariate interaction^34^ was performed with individual CPM effect as the subject effects and the rsFC during rest as the condition. This allowed the comparison of regressions between the two groups. The regressions include the relationship between seed-to-voxel rsFC (region of interest [ROI]) and CPM effect. Age and sex were included as covariates of no interest and significant interaction effects are reported at p < 0.05 Family-Wise Error (FWE) level correction to correct for multiple comparisons (with p < 0.05 two-sided false-discovery rate [FDR] correction)^34^. A priori ROIs were used as seed regions for seed-to-voxel analyses, these ROIs were areas involved with descending pain modulation, i.e. ACC, amygdala and vlPAG^1,36,37^. The left and right ACC^38^ and amygdala^39^ were acquired from the templates available in the SPM anatomy toolbox^40^. ROI maps of the vlPAG (left, right and bilateral) were provided by Ezra et al.^41,42^. All ROIs were set in MNI space. For visualisation purposes, CPM effect was plotted against the rsFC strength (Fisher transformed correlation coefficients) between ROIs showing significant associations. Pearson correlation coefficient from partial correlations (age and sex were included as covariates of no interest) between CPM effect and rsFC strength were also provided for visualisation.

## Results

### Subjects demographics

**Figure 2:**
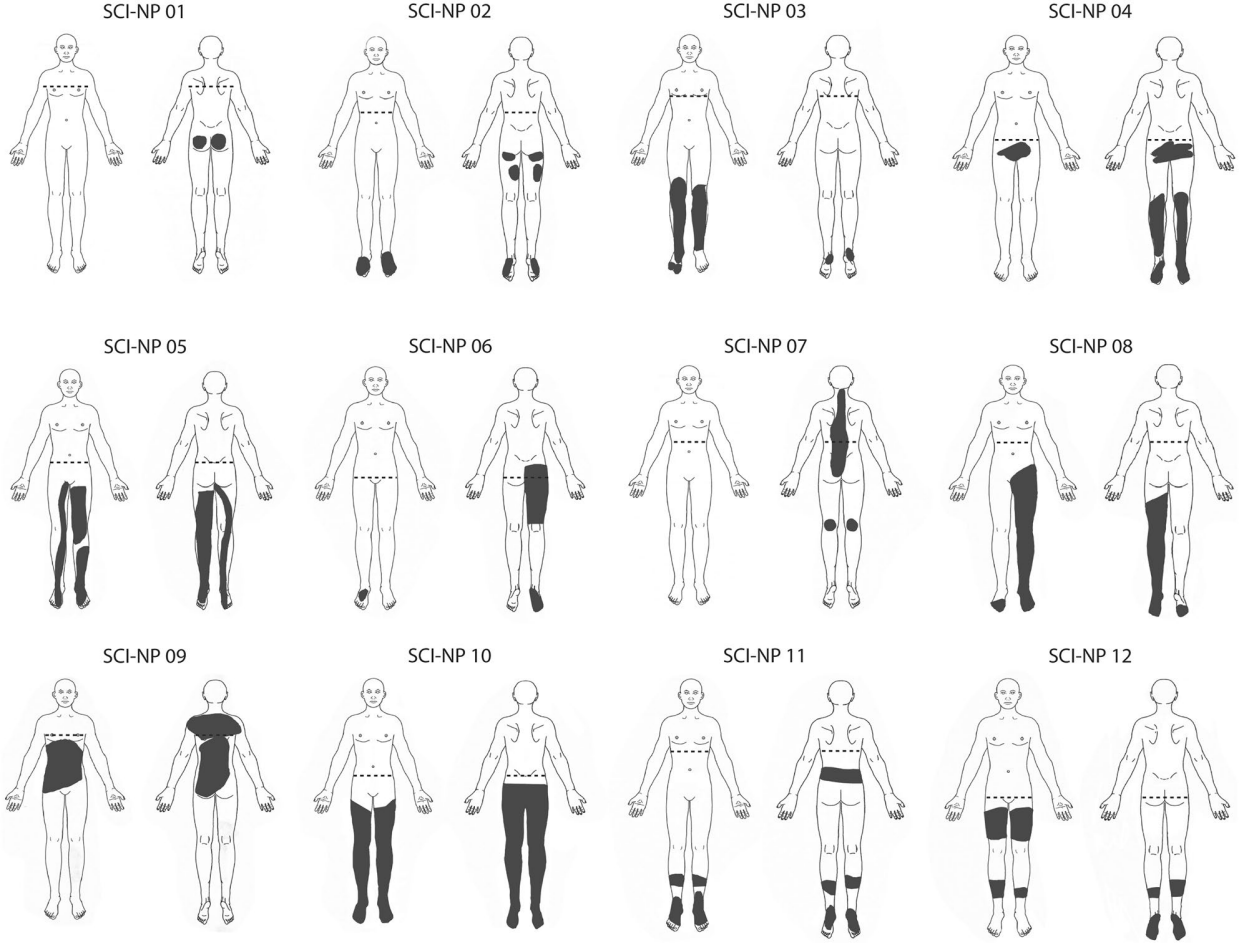
Chronic NP in SCI subjects. Diagram illustrating the area of chronic spontaneous NP in each SCI-NP subject. Dashed lines represent the neurological level of SCI and dark grey regions indicate the region of NP. NP, neuropathic pain; SCI, spinal cord injury.

One subject with SCI-NP was excluded due to impaired sensory integrity at the volar forearm above the lesion level, thus, 12 subjects with SCI-NP (Fig. 2) and 13 HC were included in the psychophysical analyses and are summarised in Table 1. No differences in subject characteristics were observed (p > 0.05) and no subject exceeded the clinical cut-off values for PCS and BDI: a total PCS score of 30 represents a clinically relevant level of catastrophising^26^ and a BDI score higher than 29 indicates severe depression^27^.

### CPM psychophysics

**Figure 3:**
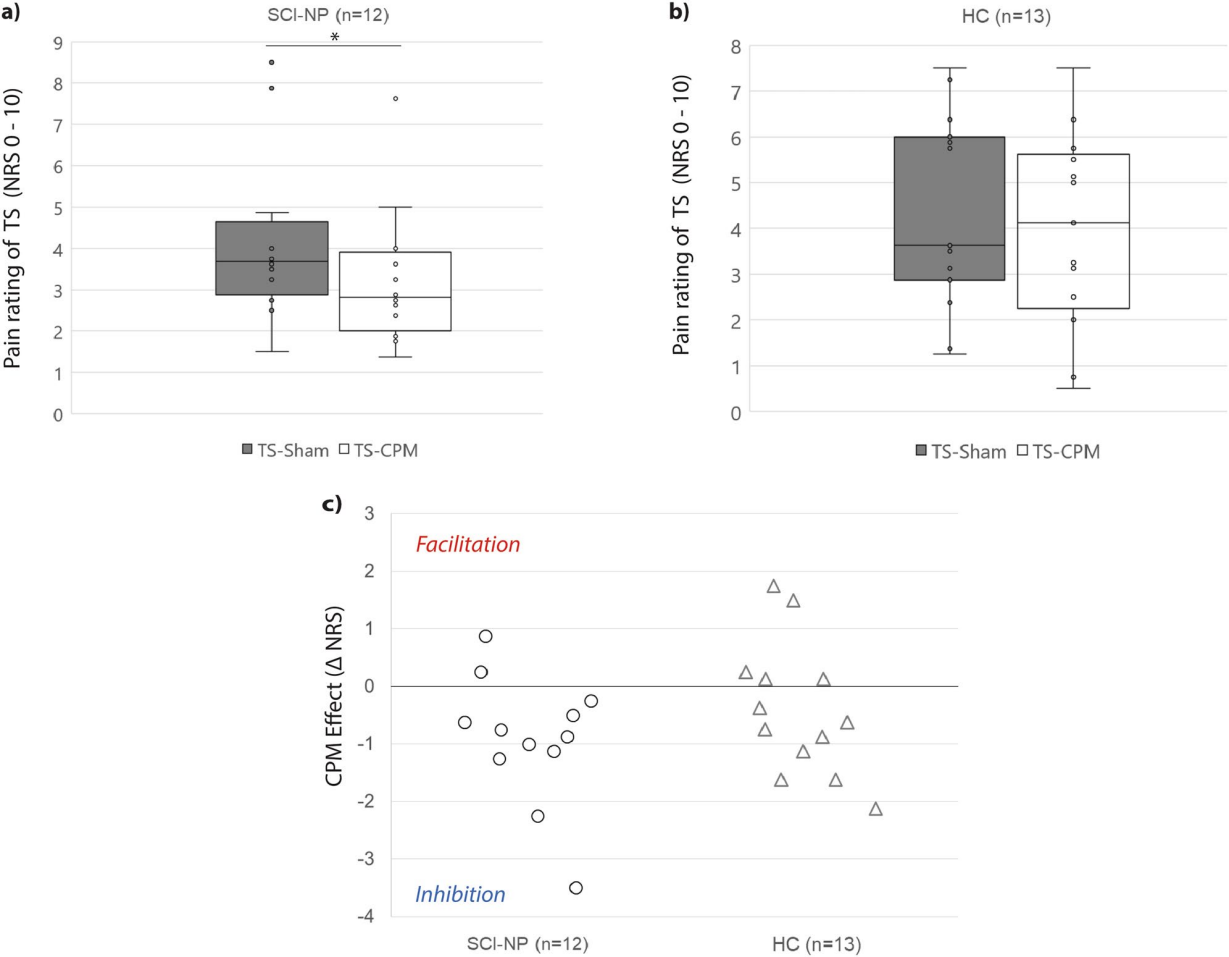
Pain ratings and CPM effect for each cohort. Psychophysical readouts. (**a**, **b**) Pain rating for TS within each condition for SCI-NP subjects and HC. (**c**) Scatter plot of individual’s CPM effect. Positive numbers describe a facilitatory CPM effect, whilst negative numbers describe an inhibitory CPM effect. Zero describes no CPM effect. CPM, conditioned pain modulation; HC, healthy controls; NRS, numerical rating scale; SCI-NP, spinal cord injury and neuropathic pain; TS-CPM, test stimulus during conditioned pain modulation; TS-Sham, test stimulus during sham condition. * Significant at p < 0.05.

Pain ratings of the TS and CS are summarised in Table 2. No subjects perceived the sham CS as noxious or painful (NRS: 0; Table 2). In the SCI-NP cohort, the pain rating of TS-CPM was lower than TS-Sham (p = 0.02) but not in HC (p = 0.22) (Fig. 3a, b). The median (range) sham-corrected CPM effect of SCI-NP subjects and HC were − 0.8 (− 3.5 to  + 0.9) and − 0.6 (− 2.1 to + 1.8), respectively (Table 1). The range of individual CPM effects are depicted in Fig. 3c. CPM effect was significantly different from 0 in the SCI-NP group (T = − 2.823; p = 0.008), whilst it was not significant in HC (T = − 1.287; p = 0.111).

**Table 2:** Psychophysical readouts.

Block (s)	SCI-NP (n = 12)				HC (n = 13)			
	TS-Sham	TS-CPM	CS	CS (Sham)	TS-Sham	TS-CPM	CS	CS (Sham)
1	6.0 ± 1.9	4.3 ± 1.5	6.5 ± 1.9	0	5.5 ± 2.1	4.7 ± 2.6	5.4 ± 2.6	0
2	4.8 ± 1.7	3.4 ± 1.4	6.3 ± 1.4	0	4.7 ± 2.4	4.1 ± 1.9	5.6 ± 2.4	0
3	4.1 ± 2.1	3.2 ± 1.6	6.4 ± 1.9	0	4.2 ± 2.3	3.9 ± 2.3	5.5 ± 2.2	0
4	3.8 ± 2.1	2.8 ± 1.6	6.2 ± 1.8	0	4.1 ± 2.0	3.8 ± 2.0	5.4 ± 2.1	0
5	3.5 ± 2.1	2.9 ± 1.7	5.8 ± 1.9	0	4.2 ± 2.2	3.7 ± 2.3	5.5 ± 2.1	0
6	3.8 ± 2.1	3.2 ± 2.1	6.3 ± 2.2	0	4.1 ± 2.2	3.9 ± 2.4	5.5 ± 2.3	0
7	3.7 ± 2.2	2.9 ± 1.9	6.2 ± 2.3	0	4.2 ± 2.0	4.0 ± 2.3	5.7 ± 2.5	0
8	3.8 ± 2.3	3.3 ± 2.4	5.8 ± 2.2	0	4.1 ± 2.1	3.6 ± 2.2	5.5 ± 2.4	0
Average	4.2 ± 2.0	3.3 ± 1.6	6.2 ± 1.8	–	4.4 ± 2.1	4.0 ± 2.1	5.5 ± 2.2	–

Mean (± SD) pain ratings of TS and CS per block. CS, conditioning stimulus; HC, healthy controls; SCI-NP, spinal cord injury and neuropathic pain; TS, test stimulus; TS-CPM, test stimulus under CPM; TS-Sham, test stimulus under Sham condition.

No difference in CPM effect (p = 0.28) was observed between the two cohorts. Additionally, individual CPM effects was not correlated with age (SCI-NP: p = 0.66; HC: p = 0.60), BDI (SCI-NP: p = 0.59; HC: p = 0.15), PCS (SCI-NP: p = 0.06; HC: p = 0.45), WDT (SCI-NP: p = 0.38; HC: p = 0.68), HPT (SCI-NP: p = 0.34; HC: p = 0.53), the rating of the CS (SCI-NP: p = 0.19; HC: p = 0.90), or time since injury in SCI-NP (p = 0.57). No difference in CPM effect was observed between SCI-NP subjects on vs off medication (SCI-NP ON medication: − 0.75 ± 1.05; SCI OFF medication: − 1.08 ± 1.27, p = 0.88).

**Figure 4:**
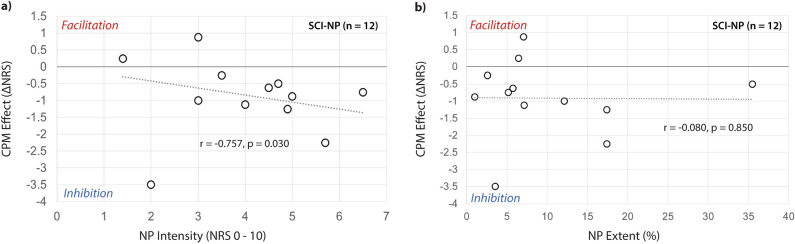
Relationship between CPM effect and NP characteristics. Partial correlations between CPM effect and NP characteristics (corrected for age and sex). (**a**, **b**) Scatter plots of NP intensity and extent with CPM effect in SCI-NP subjects (n = 12). CPM effects are described as negative and positive numbers, which indicate pain inhibition and facilitation, respectively. CPM, conditioned pain modulation; NP, neuropathic pain; NRS, numerical rating scale; TS-CPM, test stimulus under CPM; TS-Sham, test stimulus under Sham.

In SCI-NP subjects, NP intensity was negatively correlated with CPM effect (r = − 0.78, p = 0.03, Fig. 4a), indicating that the more intense the NP, the stronger the pain inhibition during CPM. CPM effect did not correlate with NP extent (r = − 0.08 p = 0.85) (Fig. 4b).

### rsFC differences in relation to CPM effect

**Figure 5:**
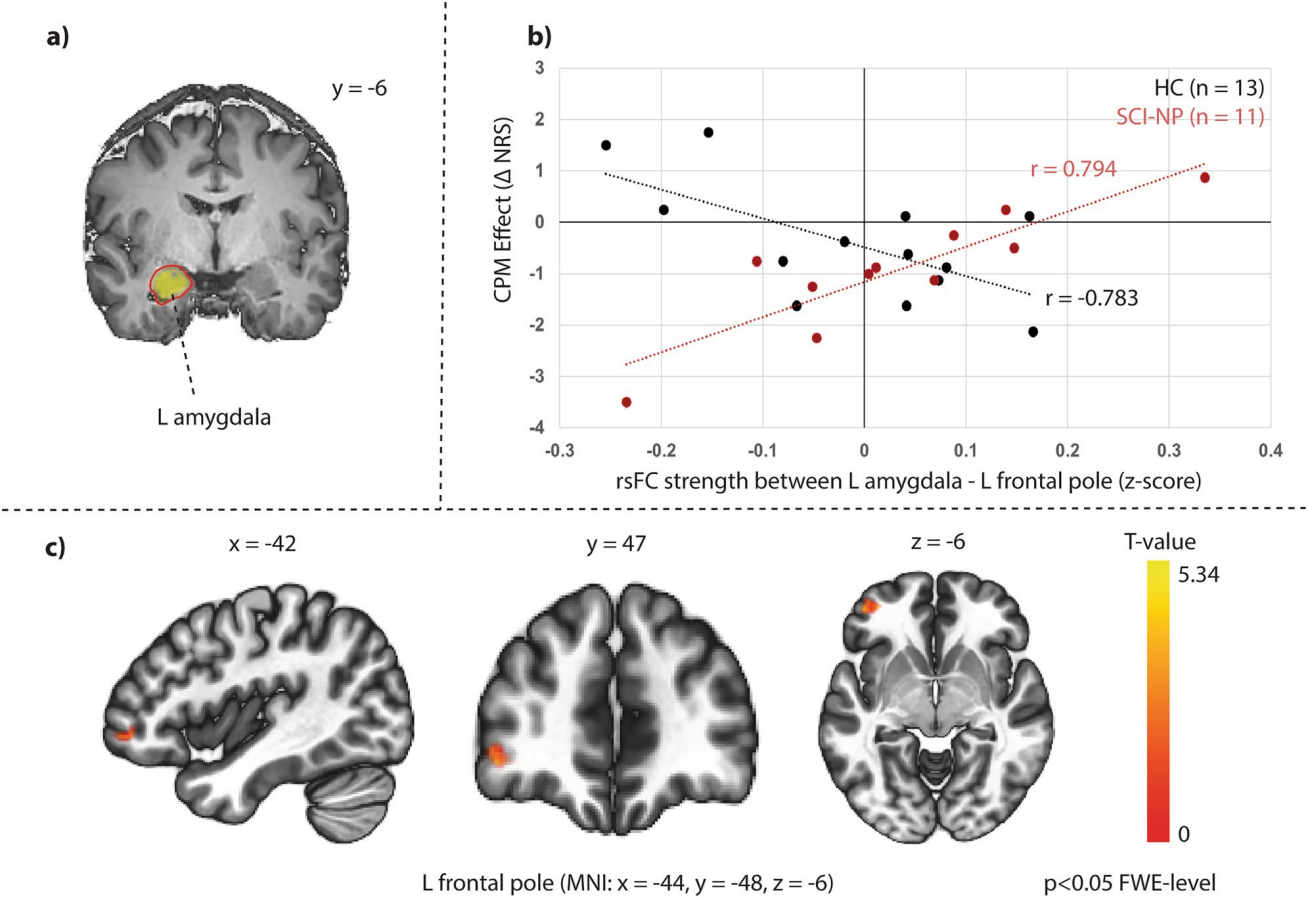
Stronger rsFC between left amygdala and left frontal pole is related to lower CPM efficiency in SCI-NP subjects. Significant group differences in correlations with CPM efficiency and rsFC between the left amygdala and left frontal pole. (**a**) Seed region for seed-to-voxel rsFC analysis. (**b**) Scatter plot representation of correlations between CPM effect and rsFC strength in each group (z-scores are Fisher transformed correlation coefficients). Individual CPM effect is described as negative and positive numbers, which indicate pain inhibition and facilitation, respectively. (**c**) Significant group interactions in relation to CPM efficiency and rsFC between seed region and clusters of the left frontal pole. MNI co-ordinates are provided and clusters were significant at p < 0.05 FWE-level corrected. CPM, conditioned pain modulation; FWE, family-wise error; HC, healthy controls; L, left; MNI, Montreal Neurological Institute; NP, neuropathic pain; NRS, numerical rating scale; R, right; SCI-NP, spinal cord injury and neuropathic pain; TS-CPM, test stimulus under CPM; TS-Sham, test stimulus under Sham condition.

**Figure 6:**
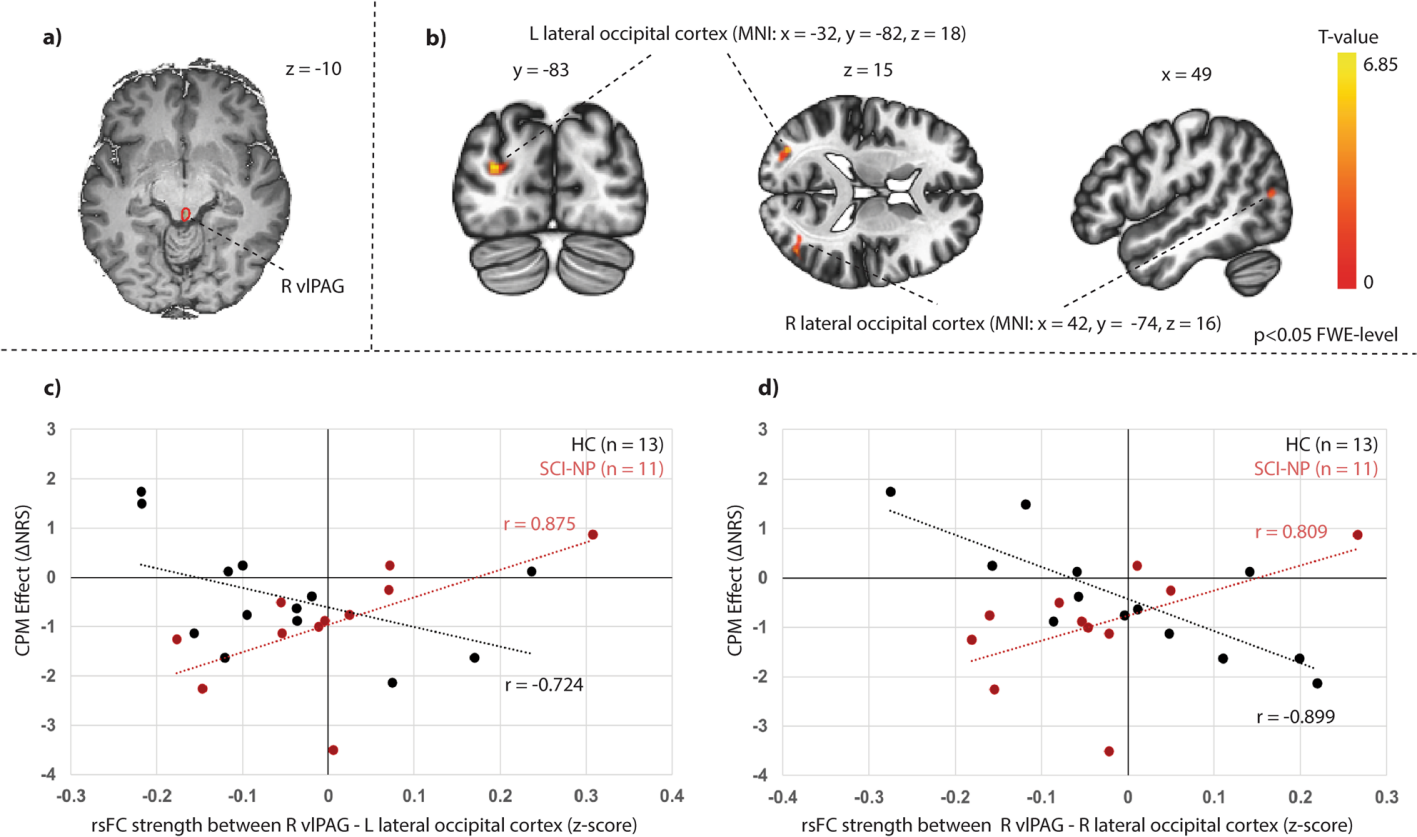
Stronger rsFC between brainstem region and visual cortex is related to lower CPM efficiency in SCI-NP subjects. Significant group differences in correlations with CPM efficiency and rsFC between the right vlPAG and bilateral occipital cortex. (**a**) Seed region for seed-to-voxel rsFC analysis. (**b**) Significant group interactions in relation to CPM efficiency and rsFC between the seed region and clusters of the lateral occipital cortex bilaterally. MNI co-ordinates are provided and clusters were significant at p < 0.05 FWE-level corrected. (**c**, **d**) Scatter plot representation of correlations between CPM effect and rsFC strength in each group (z-scores are Fisher transformed correlation coefficients). Individual CPM effect is described as negative and positive numbers, which indicate pain inhibition and facilitation, respectively. Pearson r value is also provided for visualisation. CPM, conditioned pain modulation; FWE, family-wise error; HC, healthy controls; L, left; MNI, Montreal Neurological Institute; NP, neuropathic pain; NRS, numerical rating scale; R, right; SCI-NP, spinal cord injury and neuropathic pain; TS-CPM, test stimulus under CPM; TS-Sham, test stimulus under Sham condition; vlPAG, ventrolateral periaqueductal gray.

Of the 12 SCI-NP subjects included in the psychophysical analysis, one SCI-NP subject (age: 60 years, male, CPM effect: − 0.63) showed distortion artefacts of their resting-state fMRI data and was excluded from further analyses. No subjects were excluded due to excessive head motion. The mean composite motion (maximum voxel displacement from the combined translational and rotational displacement [mean ± SD]) of each group were 0.27 ± 0.10 for HC and 0.37 ± 0.09 for SCI-NP. Thus, 11 SCI-NP subjects and 13 HC were included in the rsFC analyses. No group differences in seed-to-voxel rsFC were observed between the two cohorts when not considering CPM effect (p > 0.05 FWE-level correction). Significant interactions between group and CPM efficiency were found for rsFC between the following areas: (1) the left frontal pole (T = 3.92.; k_E_ = 82; MNI: x = − 44, y = 48, z = − 6; p-FWE = 0.03) with the left amygdala as the seed region (Fig. 4), and (2) the left (T = 6.85; k_E_ = 93; MNI: x = − 32, y = − 82, z = 18; p-FWE = 0.02) and right (T = 3.92; k_E_ = 101; MNI: x = 42, y = − 74, z = 16; p-FWE = 0.01) lateral occipital cortex with the right vlPAG as the seed region (Fig. 6). In other words, stronger rsFC between these regions were related to more efficient CPM in HC but lower CPM efficiency in SCI-NP subjects (Figs. 5, 6). No other significant interactions were observed with other ROIs as the seed region (p > 0.05 FWE-level corrected).

### Relationship between rsFC and NP characteristics

**Figure 7:**
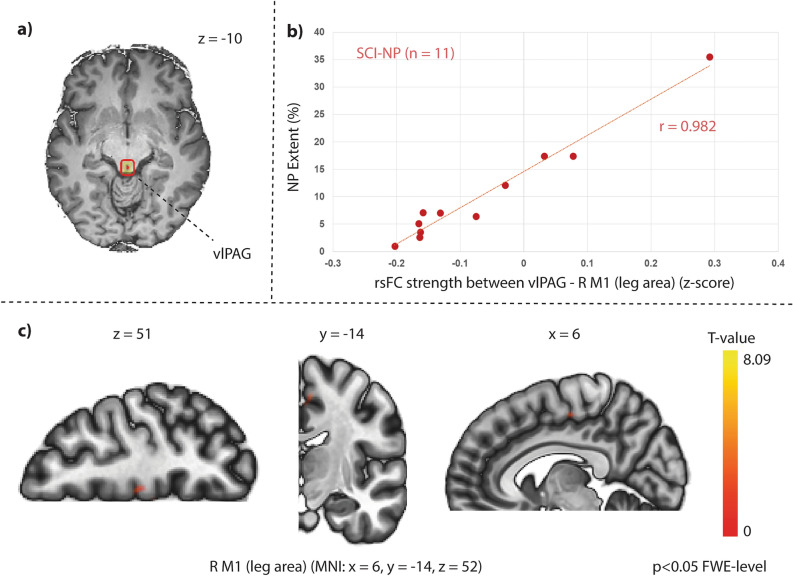
Greater NP extent in SCI-NP subjects is related to stronger rsFC between the vlPAG and leg area of the motor cortex. Relationship between NP extent and rsFC in SCI-NP subjects. (**a**) Seed region for seed-to-voxel rsFC analysis. (**b**) Scatter plot of the correlation between NP extent and rsFC strength as identified in the multiple linear regression analysis (z-scores are Fisher transformed correlation coefficients). Pearson r value is also provided for visualisation. (**c**) Significant positive association with NP extent and rsFC strength between the vlPAG and a small cluster in the right M1 leg region. MNI co-ordinates are provided and the cluster was significant at p < 0.05 FWE-level corrected. FWE, family-wise error; M1, primary motor cortex; MNI, Montreal Neurological Institute; NP, neuropathic pain; R, right; SCI-NP, spinal cord injury and neuropathic pain; vlPAG, ventrolateral periaqueductal gray.

In SCI-NP subjects, greater NP extent was positively associated with rsFC between clusters of the right primary motor cortex (M1) (T = 5.41; k_E_ = 44; MNI: x = 6, y = -14, z = 52; p-FWE = 0.02) with the vlPAG as the seed region. Specifically, the cluster was found to be within the leg area of M1 (Fig. 7).

Further, more severe NP intensity was associated with weaker rsFC between the following regions: (1) the left primary somatosensory cortex (S1)/M1 (T = 5.41; k_E_ = 98; MNI: x = − 38, y = − 12, z = 46; p-FWE < 0.001), (2) right S1/M1 (T = 5.41; k_E_ = 46; MNI: x = 30, y = − 28, z = 58; p-FWE = 0.01) and (3) right inferior frontal gyrus (pars opercularis) (T = 5.41; k_E_ = 46; MNI: x = 48, y = 10, z = 24; p-FWE = 0.01) with the left amygdala as the seed region. No other associations were reported with NP extent or intensity with any other ROI (p > 0.05 FWE-level corrected).

## Discussion

To our knowledge, this is the first study exploring the functional resting-state correlates of pain modulation in SCI-related NP. SCI-NP subjects showed preserved pain inhibition and greater CPM efficiency was related to more intense NP. Whilst there were no differences in absolute pain modulation between SCI-NP and HC, the individual CPM efficiency of SCI-NP subjects were related to different rsFC patterns as observed in other chronic pain conditions. Specifically, lower CPM efficiency was associated with stronger rsFC between regions involved with pain modulation, i.e. vlPAG and amygdala, and sensory and cognitive function, i.e. visual and frontal cortex. Greater NP extent and intensity were associated with altered rsFC between pain modulatory regions, i.e. vlPAG and amygdala, and the primary sensorimotor cortices. Taken together, these findings indicate that SCI-NP is not necessarily accompanied by dysfunctional pain inhibition, yet alterations of intrinsic connectivity at the supraspinal level and NP intensity relate to the degree and directionality of CPM.

### Functional correlates of pain modulation and NP characteristics in SCI-NP subjects

As reflected in our SCI-NP cohort, stronger rsFC between the left amygdala and left frontal cortex was related to lower CPM efficiency (Fig. 5). A similar result (enhanced rsFC between left amygdala was related to weaker CPM effect) was recently observed in chronic neck pain subjects^20^, indicating that alterations in corticolimbic connectivity could accompany pain modulatory differences and the chronification of pain^43,44^. The amygdala plays a role within the limbic circuitry involved with pain modulation^45^ and the affective-motivational components of pain, e.g. fear and anxiety^46^, whilst the frontal cortex is involved with executive function and the cognitive control of pain^47^. These regions share reciprocal anatomical connections^48,49^ and stronger rsFC between these areas have been observed in migraine^50,51^, chronic neck pain^20^ and complex regional pain syndrome^52^. The role of the left amygdala in pain modulation however remains unclear^45^, with either no effect on pain modulation, dampened pro-nociceptive function, or anti-nociceptive function^53^. In rats with NP, spontaneous activity and evoked responses in the left (central) amygdala declined six days post sciatic nerve ligation, which could account for conflicting anti- and pronociceptive functions^54^. The left amygdala also plays a role in the modulation of NP. Inactivation of the left amygdala was shown to be required to reduce mechanical allodynia induced by NP in rats^55^. Though unclear, it could be speculated that heightened rsFC between left amygdala and left frontal cortex may lead towards less inhibitory functions in SCI-NP subjects. The functional lateralisation of the amygdala during CPM in subjects with chronic pain remains unexplored which may require future studies combining CPM and task-related-fMRI.

As rsFC between the amygdala and frontal cortex was negatively correlated with CPM efficiency (i.e. stronger rsFC and greater pain inhibition) in HC, subjects with chronic pain may present an altered relationship between supraspinal rsFC and CPM efficiency^20–22^. This altered relationship may be present even though significant intact pain inhibition can be observed, as in our cohort (Fig. 3) and in other studies^21^. Further, this is substantiated by an association between lower CPM efficiency and stronger rsFC between the vlPAG and the visual cortex in SCI-NP subjects, which was inversely correlated in HC (Fig. 6). Stronger rsFC between the vlPAG with the pons has been related to lower pain inhibition (towards facilitation) in fibromyalgia^22^. Thus our findings support the notion that enhanced rsFC of pain modulatory regions, e.g. amygdala and vlPAG, to other brain areas could be contributing to lower pain inhibitory function as observed in other pain conditions^20–22^.

Whilst the vlPAG plays an essential role in pain modulation^1,2,56,57^, studies have reported an involvement of the visual system^58–60^. Visually induced analgesia has been shown to reduce pain ratings and evoked potentials induced by laser stimulation^60^. Furthermore, transcranial direct current stimulation of the visual cortex was reported to enhance analgesic effects^59^. As observed by Longo et al.^58^, visual analgesia enhanced the functional connectivity between the visual body network and the pain matrix (e.g. somatosensory cortices, insula and ACC). The activity of the visual cortex, in concert with the vlPAG, could therefore play a role in mediating stronger pain inhibitory effects in HC, but weaker inhibitory effects in SCI-NP subjects (Fig. 6).

The exploration of pain extent with digitalised drawings and neuroimaging is steadily increasing and has provided an avenue for understanding the spatial extent of pain in subjects with fibromyalgia^61^, chronic pelvic pain^62^ and SCI-related NP^63^. These studies observed that more widespread pain (i.e. greater body extent of pain) was correlated to stronger rsFC between the salience network and regions involved with sensorimotor (S1/M1)^61,62^, pain (anterior insula) and cognition function (prefrontal cortex)^61^, and within areas of the lateral pain system (thalamus and posterior insula)^63^. Our findings support that greater pain extent in chronic pain subjects is related to stronger synchronicity between supraspinal regions and could include functional changes in descending pain modulatory areas, such as the vlPAG (Fig. 7). Specifically, greater NP extent was associated with stronger rsFC between the vlPAG and a small cluster in the leg area of M1, encoding a region where NP was located in the majority of SCI-NP subjects (Fig. 2). This finding corroborates our prior study showing that aberrant brain activity is related to the extent of SCI-related NP^63^.

Previous studies have explored the neural markers of SCI-related NP intensity^63–67^, but its relationship with functional changes remains inconsistent. More severe NP intensity has been related to both weaker^65^ and stronger rsFC within, and between, pain processing and cognitive regions (e.g., insula, supplementary motor area, prefrontal cortex, parietal lobules)^63–65^, alongside increased and decreased functional plasticity of S1^67^ and M1^66^, respectively. In our current study, higher NP intensity was related to weaker rsFC between the left amygdala and bilateral sensorimotor cortices in SCI-NP subjects, which could suggest a disruption of emotional processing networks as S1 plays a role in emotional regulation possibly mediated by direct and indirect connections with the amygdala [see review:^68^].

### CPM efficiency, NP characteristics and the use of neuroimaging

Contrary to some cross-sectional studies^9,10^, the SCI-NP cohort included in this study demonstrated intact CPM efficiency (Table 1, Fig. 3) indicating that dysfunctional pain inhibition may not accompany chronic SCI-related NP. Such a significant pain inhibition has already been observed in subjects with SCI-NP^12^, diabetic neuropathy^69^ and complex regional pain syndrome^70^. In addition, our findings of preserved pain inhibition in SCI-NP are supported by recent studies^11,12^ indicating that pain inhibition using similar CPM paradigms could remain intact above the level of neurological injury. Further, higher NP intensity was related to greater pain inhibition and there was no relationship with NP extent (Fig. 4). Though this finding contrasts to prior studies observing that greater number of painful body regions^10^ and higher NP intensity are correlated with weaker pain inhibition^9^, it is not unexpected according to current evidence. In line with our findings, Gagné et al.^11^, observed that SCI-NP subjects with higher NP intensity presented with greater pain inhibition. In addition, such a positive correlation between self-reported spontaneous pain intensity and CPM capacity was also recently reported in subjects with painful diabetic neuropathy^71^. Together these findings substantiate the speculation that more severe NP is related to greater anti-nociceptive functions via descending pain control^11,69–71^.

One rationale for why intense pain might lead to more efficient pain inhibition can be observed in animal models of inflammatory or NP. In these models, mechanical or thermal CS of the affected hind paw induced an enhanced diffuse noxious inhibitory control (DNIC) response on the activity of the trigeminal convergent neurons^72^. However, this rationale may not explain the findings of the current study as our CPM paradigm was implemented in a non-painful area above the level of lesion (Fig. 1). A study by Bouhassira et al.^73^ demonstrated that mechanical stimulation of allodynic area inhibits nociceptive reflex and accompanying painful sensations in subjects with traumatic peripheral nerve injury. The extent of this inhibition was comparable with the CPM efficiency even when the CS was applied to the normal, non-painful limb^73^. This study suggests that spontaneous ongoing pain may result from other abnormal central processes independent of those sub-serving DNIC as pain inhibition in these subjects was clearly intact. Therefore, the pathophysiology of spontaneous NP present in our current SCI-NP cohort may be independent of CPM processes (even though they are correlated) and pain inhibition can remain intact above the level of lesion. This is consistent with a longitudinal study by Gagné et al.^11^ demonstrating that impaired CPM may not be causative of NP after SCI. In SCI-NP subjects, pain inhibition was also lower at discharge compared to admission and greater pain inhibition was related to higher NP intensity at admission. This indicates that NP could be defensively boosting or engaging CPM and increase anti-nociceptive functions in the initial phases of SCI-NP, which may also be present in the chronic phase as indicated in our findings (Fig. 4). Taken together, efficient CPM may be observed in SCI-NP subjects when tested in sensory intact regions and more severe NP could be engaging anti-nociceptive functions though it remains unclear whether the mechanisms of CPM are directly related to the presence of chronic NP.

Further, though psychological factors have been associated with CPM efficiency in chronic pain, e.g. greater PCS was related to weaker pain inhibition in chronic low back pain^74^, there were no observable relationships between PCS, or BDI, with CPM efficiency in our SCI-NP cohort.

The null finding related to no overall pain inhibition in our HC cohort, is not exceptional to our study^22,75,76^. One possible explanation for this observation might be related to the age of our HC cohort (mean age: 52.9 years). Indeed, it is a known tendency that CPM efficiency decreases with increasing age^71,77–79^, yet, the associations between rsFC and CPM efficiency in HC are aligned with previous observations (i.e. stronger rsFC and greater pain inhibition)^21,22^. However, the current CPM paradigm has been implemented in a previous study of 40 healthy subjects and demonstrated pain inhibition overall (mean ± SD: − 0.57 ± 1.15)^24^. The variability of CPM effect has also been shown in pain-free subjects with partial pain facilitation being considered a normal finding^80^. The variation in individual pain inhibition or facilitation in HC may also be related to the strength of rsFC between top-down (vlPAG and M1) and bottom-up pain-processing regions (amygdala and posterior insula), respectively^24^. Therefore, this study supports accumulating evidence that distinctions of CPM efficiency between chronic pain subjects and pain-free subjects may be identified with neuroimaging methods^20–22,24,81,82^.

### Limitations

Firstly, a cohort of SCI subjects without NP was not included and the SCI-NP cohort is small, and though comparable numbers of subjects with fibromyalgia were included in a similar study (Harper et al.^22^), the generalisability of the findings presented here might be limited. Moreover, six SCI-NP subjects were on stable doses of pain medication and due to ethical reasons, it was not possible to examine these subjects off medication within a period of several days/weeks. However, our cohorts were well-matched in demographics (Table 1). Secondly, our CPM paradigm was adapted to perform within the limits of an MRI scanner and may deviate from recommendations of CPM testing^8^, although the current CPM paradigm has been implemented in a previous study of 40 HC and demonstrated pain inhibition overall^24^. Further, the application of the CS was performed in parallel with the TS during CPM, hence the inhibitory effects may be in part due to distraction effects. Distraction have been shown to add a significant amount of pain inhibition during CPM^83^. Though the use of a sham condition, may account for the potential distraction effect. Although the conditions were randomised, the gap between each condition was five-minutes which could contain some carry-over effects which can influence the CPM effect^84^. However, prior CPM studies have reported few or no carry-over effects with five-minute intervals^85^. The MRI analysis was not optimised for lower brainstem regions and indeed, optimising these parameters allows more precise examinations of lower brainstem regions (e.g. pons and medulla) activity in CPM^76^. Future studies that wish to disentangle brainstem mechanisms with rsFC should consider optimising parameters solely on brainstem regions. Finally, the acquisition time of the resting-state fMRI is five minutes, and though this duration is able to acquire stable estimates of intrinsic connectivity networks^84,85^, longer acquisition times (e.g. up to 13 min) could increase the reliability of functional connectivity^86^. As the findings of this study are associations of subjective readouts with rsFC measures, a causative relationship between the brain regions and inhibitory capacity cannot be inferred. To provide this insight, future studies would need to investigate the neural activity of SCI-NP subjects by combining CPM and task-related fMRI.

## Conclusions

Our findings highlight that SCI-related NP is not necessarily accompanied by dysfunctional pain inhibition, yet SCI-NP subjects can be differentiated from HC by an abnormal relationship between the intrinsic connectivity of pain-related regions and individual pain modulatory capacity. This study also supports that the variability of CPM seen in pain cohorts and HC are related to their underlying neural plasticity of distinctive pain-related regions. However, studies with larger cohorts of SCI subjects (including those without NP) are necessary to corroborate our findings. Longitudinal studies will also be required to disentangle the interplay and cause-and-effect relationship between SCI-NP development alongside alterations in rsFC and pain modulation. Complementing CPM testing with neuroimaging methods can improve the understanding of CPM variability and provide an avenue to identify neural correlates of endogenous pain modulation in patients with chronic pain.

## Data Availability

The data that support the findings of this study are available from the corresponding author upon reasonable request.
